# Supplementary material for: Nonreciprocity and magnetic-free isolation based on optomechanical interactions
Source: Nat Commun. 2016 Nov 29;7:13662. doi: 10.1038/ncomms13662 (PMC5141342; doi:10.1038/ncomms13662)
Supplement: Supplementary Information — Supplementary Notes 1-6 and Supplementary References [file ncomms13662-s1.pdf]

Supplementary Note 1: General requirements of non-reciprocity in a two-mode optical system

In general, the temporal-evolution equations of a two-mode system can be described via [1]:

$$\frac{d}{dt}\begin{pmatrix}\delta a_1\\ \delta a_2\end{pmatrix} = \mathrm{i}\mathcal{M}\begin{pmatrix}\delta a_1\\ \delta a_2\end{pmatrix} + D^{\mathrm{T}}\begin{pmatrix}\delta s_1^+\\ \delta s_2^+\end{pmatrix}, \tag{1}$$

$$\begin{pmatrix}\delta s_1^-\\ \delta s_2^-\end{pmatrix} = C\begin{pmatrix}\delta s_1^+\\ \delta s_2^+\end{pmatrix} + D\begin{pmatrix}\delta a_1\\ \delta a_2\end{pmatrix}, \tag{2}$$

where $\mathcal{M}$ is the linear evolution operator for the two optical modes, the matrix D describes the coupling between the two ports and optical modes and C describes the direct scattering path between the two ports. Note that δs_j^+ and δs_j^- are normalized such that $|\delta s_j^+|^2$ is the input photon flux in channel j. In writing these equations, we have enforced the optical modes to each satisfy reciprocity, in the sense that the in-and out-coupling rates of a particular mode and port are equal, thus the coupling is described through the same matrix D (and its transpose) in Supplementary Equations 1 and 2 [1]. In the frequency domain $\big((a[\omega], s[\omega]) = \int (a(t), s(t)) \exp(\mathrm{i}\omega t)\, dt\big)$, Supplementary Equation 1 can be written as

$$-\mathrm{i}(M+\omega I)\begin{pmatrix}\delta a_1\\ \delta a_2\end{pmatrix} = D^{\mathrm{T}}\begin{pmatrix}\delta s_1^+\\ \delta s_2^+\end{pmatrix}, \tag{3}$$

where I represents the 2×2 identity matrix. The scattering matrix, defined as

$$\begin{pmatrix}\delta s_1^-\\ \delta s_2^-\end{pmatrix} = S\begin{pmatrix}\delta s_1^+\\ \delta s_2^+\end{pmatrix}, \tag{4}$$

is now directly obtained to be

$$S = C + \mathrm{i}D(M+\omega I)^{-1}D^{\mathrm{T}}. \tag{5}$$

A direct calculation of the off-diagonal scattering elements (forward and backward transmission coefficients) reveals that

$$S_{12} = c_{12} + \mathrm{i}\frac{(m_{11}+\omega)d_{12}d_{22} - m_{12}d_{11}d_{22} - m_{21}d_{12}d_{21} + (m_{22}+\omega)d_{11}d_{21}}{\det(M+\omega I)}, \tag{6}$$

$$S_{21} = c_{21} + \mathrm{i}\frac{(m_{11}+\omega)d_{12}d_{22} - m_{12}d_{12}d_{21} - m_{21}d_{11}d_{22} + (m_{22}+\omega)d_{11}d_{21}}{\det(M+\omega I)}. \tag{7}$$

For a symmetric (reciprocal) scattering matrix C, the contrast between forward and backward transmission coefficients now reads

$$S_{21} - S_{12} = \frac{\mathrm{i}\det(D)(m_{12}-m_{21})}{\det(M+\omega I)}. \tag{8}$$

From this formula, it becomes immediately clear that the necessary condition for breaking reciprocity is to ensure $\det(D)\times(m_{12}-m_{21}) \neq 0$, which requires: (a) $\det(D)\neq 0$, and (b) $m_{12}\neq m_{21}$. As discussed in the main text, the first condition simply requires D to be a full rank matrix, which is expected as in the case of a non-full-rank D both optical modes are symmetrically coupled to the two ports, thus preserving reciprocity.

Supplementary Note 2: Connection between Coupled-Mode Theory and cavity QED

In this section we investigate the connection between Coupled Mode Theory (CMT) — well-established in the optics and engineering literature [1] — and the quantum optics input/output formalism that is widely used in CQED [2, 3, 4]. This regards principally equations (3) and (4) of our manuscript. In the following, we show how both formalisms are mathematically related, and how it is possible to transform fields and matrices to move from one picture to the other. This transformation involves a redefinition of the input (or output) fields.

In a CQED approach, the input/output relation is conventionally written as

$$\mathbf{s}^{-} = \tilde{\mathbf{s}}^{+} + D\,\mathbf{a}, \tag{9}$$

where for a two mode system

$$\mathbf{s}^{-} = \begin{pmatrix} s_1^{-} \\ s_2^{-} \end{pmatrix}, \quad \tilde{\mathbf{s}}^{+} = \begin{pmatrix} \tilde{s}_1^{+} \\ \tilde{s}_2^{+} \end{pmatrix} \text{ and } \mathbf{a} = \begin{pmatrix} a_1 \\ a_2 \end{pmatrix} \tag{10}$$

denote the output, input and intracavity field operators, respectively. This is for example detailed in [3], section 5.3. Note that in Supplementary Equation (9) an explicit choice for the phase relation between input and output fields is made (sometimes taken with an extra minus sign [4], with similar result), as in absence of the cavity they are related by the identity operator. Instead, in CMT a specific choice of this phase is avoided by introducing the C matrix operator such that

$$\mathbf{s}^{-} = C\,\mathbf{s}^{+} + D\,\mathbf{a}, \tag{11}$$

which is Eq. (4) in our manuscript. Note that the relation $\tilde{\mathbf{s}}^{+} = C\mathbf{s}^{+}$ thus allows to transform outputs between the two formalisms. Applying this transformation to Eq. (3) from our manuscript,

$$\frac{d\mathbf{a}}{dt} = \mathrm{i}\mathcal{M}\mathbf{a} + D^{\mathrm{T}}\,\mathbf{s}^{+}, \tag{12}$$

using the relation $CD^{*} = -D$ (obtained from time-reversal symmetry in [1]), we obtain

$$\begin{aligned} \frac{d\mathbf{a}}{dt} &= \mathrm{i}\mathcal{M}\,\mathbf{a} - (CD^{*})^{\mathrm{T}}\;\mathbf{s}^{+} && (13)\\ &= \mathrm{i}\mathcal{M}\,\mathbf{a} - D^{\dagger}C\,\mathbf{s}^{+} && (14)\\ &= \mathrm{i}\mathcal{M}\,\mathbf{a} - D^{\dagger}\,\tilde{\mathbf{s}}^{+}. && (15) \end{aligned}$$

This is precisely equivalent to the expression often used in CQED. We thus conclude that both formalisms differ only in the sense that CQED explicitly chooses a convention for the port description, fixing the phase of the incoming waves, while CMT does not.

The CMT formalism has the benefit that it allows to associate s_j^{+} and s_j^{-} with incoming and outgoing waves in the same physical port. Considering the CQED approach (Supplementary Equation 9) and a simple waveguide, s_1^{+} and s_1^{-} necessarily describe waves in different physical ports. To study nonreciprocity, it is more insightful to have these waves in the same port, as nonreciprocity is then always related to a difference of the off-diagonal elements of the scattering matrix, regardless of system choice.

Supplementary Note 3: Breaking the symmetry of the evolution matrix through optomechanical coupling

Here we consider the optical modes to have generally different resonant frequencies $\omega_{1,2}$ and energy decay rates κ_1 and κ_2. In addition, we describe the mechanical mode by its resonance frequency $\mathit{\Omega}_{\mathrm{m}}$ and loss rate $\mathit{\Gamma}_{\mathrm{m}}$. Starting from the linearized Hamiltonian in equation (2) of the main text, the equations of motion for the photon and phonon annihilation operators can be written as:

$$\frac{d}{dt}\begin{pmatrix}\delta a_1\\ \delta a_2\end{pmatrix} = \mathrm{i}\mathit{\Theta}\begin{pmatrix}\delta a_1\\ \delta a_2\end{pmatrix} + \mathrm{i}\begin{pmatrix}g_1(b+b^\dagger)\\ g_2(b+b^\dagger)\end{pmatrix} + D^{\mathrm{T}}\begin{pmatrix}\delta s_1^+\\ \delta s_2^+\end{pmatrix}, \tag{16}$$

$$\frac{d}{dt}b = (-\mathrm{i}\mathit{\Omega}_{\mathrm{m}} - \mathit{\Gamma}_{\mathrm{m}}/2)\,b + \mathrm{i}(g_1^*\delta a_1 + g_1\delta a_1^\dagger + g_2^*\delta a_2 + g_2\delta a_2^\dagger) + +\sqrt{\mathit{\Gamma}_{\mathrm{m}}}b_{\mathrm{in}}, \tag{17}$$

where in this relation $g_{1,2}$ represent the enhanced optomechanical coupling rates defined as $g_1 = G_1 x_{\mathrm{zpf}}\alpha_1$ and $g_2 = G_2 x_{\mathrm{zpf}}\alpha_2$, while $\mathit{\Theta}$ is a 2×2 matrix which describes the evolution of the optical modes in the absence of the optomechanical interactions and in the absence of port excitations. For completeness Supplementary Equation 17 contains the coupling between the mechanical resonator and the mechanical heat bath (rightmost term). As shown in the methods section, this term can be set to zero for the experimental parameters studied here. Likewise, quantum fluctuations of optical inputs are neglected in Supplementary Equation 16. In general $\mathit{\Theta}$ can be written as $\mathit{\Theta} = \mathit{\Omega} + \mathrm{i}K$ where the matrix $\mathit{\Omega}$ contains the resonance frequencies and the mutual coupling of the two modes on diagonal and off-diagonal elements respectively. The matrix $K = K_0 + K_{\mathrm{e}}$, on the other hand, represents the total losses of the two modes $\kappa_{1,2} = \kappa_{0_{1,2}} + \kappa_{\mathrm{e}_{1,2}}$ which includes the intrinsic losses ($\kappa_{0_{1,2}}$) due to absorption and scattering as well as the out-coupling losses ($\kappa_{\mathrm{e}_{1,2}}$) due to the leakage of the optical modes into the two ports. Even though in general $\mathit{\Theta}$ is not diagonal, it is always possible to choose a proper eigenmode basis such that $\mathit{\Theta}$ becomes diagonal (see Supplementary Note 4). In that case, it can be written as

$$\mathit{\Theta} = \begin{pmatrix}\bar{\Delta}_1 + \mathrm{i}\frac{\kappa_1}{2} & 0\\ 0 & \bar{\Delta}_2 + \mathrm{i}\frac{\kappa_2}{2}\end{pmatrix}, \tag{18}$$

where $\bar{\Delta}_1 = \omega_{\mathrm{control}} - \bar{\omega}_1 + G_1\bar{x}$, $\bar{\Delta}_2 = \omega_{\mathrm{control}} - \bar{\omega}_2 + G_2\bar{x}$ represent the modified frequency detunings of the two optical modes from the driving lasers. In the frequency domain, Supplementary Equations 16 and 17 become

$$\mathrm{i}\begin{pmatrix}\mathit{\Sigma}_{\mathrm{o}_1} & 0\\ 0 & \mathit{\Sigma}_{\mathrm{o}_2}\end{pmatrix}\begin{pmatrix}\delta a_1\\ \delta a_2\end{pmatrix} + \mathrm{i}\begin{pmatrix}g_1(b+b^\dagger)\\ g_2(b+b^\dagger)\end{pmatrix} + D^{\mathrm{T}}\begin{pmatrix}\delta s_1^+\\ \delta s_2^+\end{pmatrix} = 0, \tag{19}$$

$$\mathrm{i}\mathit{\Sigma}_{\mathrm{m}}^+\,b + \mathrm{i}(g_1^*\delta a_1 + g_1\delta a_1^\dagger + g_2^*\delta a_2 + g_2\delta a_2^\dagger) = 0, \tag{20}$$

where we introduce a shorthand notation for the Fourier transformed creation operators $\delta a^\dagger = (\delta a^\dagger)[\omega] = (\delta a[-\omega])^\dagger$, and defined the inverse susceptibilities

$$\mathit{\Sigma}_{\mathrm{m}}^{\pm} \equiv \omega \mp \mathit{\Omega}_{\mathrm{m}} + \mathrm{i}\frac{\mathit{\Gamma}_{\mathrm{m}}}{2}; \quad \mathit{\Sigma}_{\mathrm{o}_{1,2}} \equiv \omega + \bar{\Delta}_{1,2} + \mathrm{i}\frac{\kappa_{1,2}}{2} \tag{21}$$

Using Supplementary Equations 19 and 20 along with their Hermitian conjugate, it is straightforward to show that

$$\mathrm{i}\begin{pmatrix}\Sigma_{\mathrm{o}_1} & 0\\ 0 & \Sigma_{\mathrm{o}_2}\end{pmatrix}\begin{pmatrix}\delta a_1\\ \delta a_2\end{pmatrix} + \mathrm{i}\left(\frac{1}{\Sigma_{\mathrm{m}}^-}-\frac{1}{\Sigma_{\mathrm{m}}^+}\right)\begin{pmatrix}|g_1|^2 & g_1 g_2^*\\ g_1^* g_2 & |g_2|^2\end{pmatrix}\begin{pmatrix}\delta a_1\\ \delta a_2\end{pmatrix}$$
$$+\mathrm{i}\left(\frac{1}{\Sigma_{\mathrm{m}}^-}-\frac{1}{\Sigma_{\mathrm{m}}^+}\right)\begin{pmatrix}g_1^2 & g_1 g_2\\ g_1 g_2 & g_2^2\end{pmatrix}\begin{pmatrix}\delta a_1^\dagger\\ \delta a_2^\dagger\end{pmatrix} + D^{\mathrm{T}}\begin{pmatrix}\delta s_1^+\\ \delta s_2^+\end{pmatrix} = 0. \tag{22}$$

For the remainder we will consider operation in the resolved side-band regime, which allows ignoring the non-resonant terms involving $a_{1,2}^\dagger$ in Supplementary Equation 22, such that it simplifies to

$$\mathrm{i}\left[\begin{pmatrix}\Sigma_{\mathrm{o}_1} & 0\\ 0 & \Sigma_{\mathrm{o}_2}\end{pmatrix} + \mathrm{i}\left(\frac{1}{\Sigma_{\mathrm{m}}^-}-\frac{1}{\Sigma_{\mathrm{m}}^+}\right)\begin{pmatrix}|g_1|^2 & g_1 g_2^*\\ g_1^* g_2 & |g_2|^2\end{pmatrix}\right]\begin{pmatrix}\delta a_1\\ \delta a_2\end{pmatrix} + D^{\mathrm{T}}\begin{pmatrix}\delta s_1^+\\ \delta s_2^+\end{pmatrix} = 0. \tag{23}$$

Given that we are particularly interested in probe signals that are detuned from the control by approximately the mechanical resonance frequency ($|\omega \mp \Omega_{\mathrm{m}}| \ll \Omega_{\mathrm{m}}$), one can always neglect one of the two terms involving mechanical susceptibilities $1/\Sigma_{\mathrm{m}}^{\pm}$. For $\bar{\Delta}_j \approx \mp\Omega_{\mathrm{m}}$, the frequency-domain evolution matrix M can now be found from Supplementary Equation 23 to be

$$M = \begin{pmatrix}\bar{\Delta}_1 + \mathrm{i}\frac{\kappa_1}{2} & 0\\ 0 & \bar{\Delta}_2 + \mathrm{i}\frac{\kappa_2}{2}\end{pmatrix} \mp \frac{1}{\Sigma_{\mathrm{m}}^{\pm}}\begin{pmatrix}|g_1|^2 & g_1 g_2^*\\ g_1^* g_2 & |g_2|^2\end{pmatrix}. \tag{24}$$

Clearly, in order to break the symmetry of M, which is a necessary condition for breaking the reciprocity of the system, one needs to enforce $g_1 g_2^* \neq g_1^* g_2$, thus requiring $\Delta\phi = \arg(g_2) - \arg(g_1) \neq n\pi$ where $n \in \mathbb{N}$. The resulting scattering matrix reads

$$S = C + \mathrm{i}D(M+\omega I)^{-1}D^{\mathrm{T}} = C + \mathrm{i}D\begin{pmatrix}\Sigma_{\mathrm{o}_1} \mp \frac{|g_1|^2}{\Sigma_{\mathrm{m}}^{\pm}} & \mp\frac{g_1 g_2^*}{\Sigma_{\mathrm{m}}^{\pm}}\\ \mp\frac{g_1^* g_2}{\Sigma_{\mathrm{m}}^{\pm}} & \Sigma_{\mathrm{o}_2} \mp \frac{|g_2|^2}{\Sigma_{\mathrm{m}}^{\pm}}\end{pmatrix}^{-1} D^{\mathrm{T}}. \tag{25}$$

Using Supplementary Equation 8, the difference in transmission $S_{21} - S_{12}$ is directly given as

$$S_{21} - S_{12} = 2\sin\Delta\phi\frac{\det D}{\sqrt{\kappa_1\kappa_2}}\frac{\mp 2\sqrt{\mathcal{C}_1\mathcal{C}_2}}{(\delta_\pm + \mathrm{i})(\delta_1+\mathrm{i})(\delta_2+\mathrm{i}) \mp (\mathcal{C}_2(\delta_1+\mathrm{i}) + \mathcal{C}_1(\delta_2+\mathrm{i}))}, \tag{26}$$

where again the upper and lower signs relate to red ($\bar{\Delta}_{1,2} \approx -\Omega_{\mathrm{m}}$) and blue ($\bar{\Delta}_{1,2} \approx +\Omega_{\mathrm{m}}$) detuned regimes respectively, and we have defined

$$\mathcal{C}_j \equiv \frac{4|g_j|^2}{\kappa_j \Gamma_{\mathrm{m}}}, \quad \delta_\pm \equiv \frac{\omega \mp \Omega_{\mathrm{m}}}{\Gamma_{\mathrm{m}}/2}, \quad \delta_j \equiv \frac{\omega + \bar{\Delta}_j}{\kappa_j/2}. \tag{27}$$

Alternatively, one can obtain S_{12} and S_{21} separately, which are given by:

$$S_{12} = c_{12} + \mathrm{i}\frac{2A \pm 2\sqrt{\mathcal{C}_1\mathcal{C}_2}(d_{12}d_{21}e^{\mathrm{i}\Delta\phi} + d_{11}d_{22}e^{-\mathrm{i}\Delta\phi}}{\sqrt{\kappa_1\kappa_2}[(\delta_\pm+\mathrm{i})(\delta_1+\mathrm{i})(\delta_2+\mathrm{i}) \mp (\mathcal{C}_2(\delta_1+\mathrm{i}) + \mathcal{C}_1(\delta_2+\mathrm{i}))]}, \tag{28}$$

$$S_{21} = c_{21} + \mathrm{i}\frac{2A \pm 2\sqrt{\mathcal{C}_1\mathcal{C}_2}(d_{11}d_{22}e^{\mathrm{i}\Delta\phi} + d_{12}d_{21}e^{-\mathrm{i}\Delta\phi}}{\sqrt{\kappa_1\kappa_2}[(\delta_\pm+\mathrm{i})(\delta_1+\mathrm{i})(\delta_2+\mathrm{i}) \mp (\mathcal{C}_2(\delta_1+\mathrm{i}) + \mathcal{C}_1(\delta_2+\mathrm{i}))]}, \tag{29}$$

where

$$A = d_{11}d_{21}\sqrt{\frac{\kappa_2}{\kappa_1}}[(\delta_\pm+\mathrm{i})(\delta_2+\mathrm{i}) \mp \mathcal{C}_2] + d_{12}d_{22}\sqrt{\frac{\kappa_1}{\kappa_2}}[(\delta_\pm+\mathrm{i})(\delta_1+\mathrm{i}) \mp \mathcal{C}_1], \tag{30}$$

Note that these expressions are general in the sense that they are valid regardless of the way the modes are coupled to the ports. In Supplementary Note 6 we will consider a specific implementation.

Supplementary Note 4: Diagonalization of the optical evolution matrix

Here we show that in general it is always possible to find a proper eigenbasis that diagonalizes the evolution matrix of an optical system. To show this, consider an optical system described through the coupled mode equations:

$$\frac{d}{dt}\mathbf{a} = \mathrm{i}\Theta\mathbf{a} + D^{\mathrm{T}}\mathbf{s}_+, \tag{31}$$

$$\mathbf{s}_- = C\mathbf{s}_+ + D\mathbf{a}, \tag{32}$$

where $\mathbf{a} = (a_1\ a_2)^{\mathrm{T}}$ represents the modal amplitude of the two modes and $\mathbf{s}_\pm = (s_1^\pm\ s_2^\pm)^{\mathrm{T}}$ represents the vector of inputs/outputs at two ports. The evolution matrix Θ can in general be written in form of:

$$\Theta = \begin{pmatrix} \Delta_1 & \mu \\ \mu & \Delta_2 \end{pmatrix} + \mathrm{i}/2 \begin{pmatrix} \kappa_1 & \kappa_r \\ \kappa_r & \kappa_2 \end{pmatrix} \tag{33}$$

where in this relation $\Delta_{1,2}$ represent the frequency detunings of the two modes with respect to a central resonance frequency, μ represents the mutual coupling and κ_1, κ_2 and κ_r are the optical losses due to intrinsic losses and leakage to ports as well as mutual coupling. Defining X_{R} and X_{L} as two matrices with the right and left eigenvectors of Θ as their columns and rows respectively, one can write [5]:

$$\Theta X_{\mathrm{R}} = X_{\mathrm{R}}\Lambda, \tag{34}$$

$$X_{\mathrm{L}}\Theta = \Lambda X_{\mathrm{L}}, \tag{35}$$

where Λ is the diagonal matrix of eigenvalues. Given that Θ is symmetric, the left and right eigenvectors are related via $X_{\mathrm{L}} = X_{\mathrm{R}}^{\mathrm{T}}$. On the other hand, one can show that $X_{\mathrm{L}}X_{\mathrm{R}} = X_{\mathrm{R}}X_{\mathrm{L}}$ is diagonal. Therefore through a proper normalization of the eigenvectors one can write $X_{\mathrm{R}}^{\mathrm{T}}X_{\mathrm{R}} = X_{\mathrm{R}}X_{\mathrm{R}}^{\mathrm{T}} = I$, thus X_{R} is an orthogonal matrix. Supplementary Equations 31 and 32 can now be written as:

$$\frac{d}{dt}X_{\mathrm{R}}\,\mathbf{a} = \mathrm{i}X_{\mathrm{R}}\,\Theta X_{\mathrm{R}}^{\mathrm{T}}X_{\mathrm{R}}\,\mathbf{a} + X_{\mathrm{R}}D^{\mathrm{T}}\mathbf{s}_+, \tag{36}$$

$$\mathbf{s}_- = C\mathbf{s}_+ + DX_{\mathrm{R}}^{\mathrm{T}}X_{\mathrm{R}}\,\mathbf{a}, \tag{37}$$

which can be written in form of Supplementary Equations 31 and 32 as

$$\frac{d}{dt}\,\mathbf{a}' = \mathrm{i}\Theta'\,\mathbf{a}' + D'^{\mathrm{T}}\mathbf{s}_+, \tag{38}$$

$$\mathbf{s}_- = C\mathbf{s}_+ + D'\,\mathbf{a}', \tag{39}$$

Here, $\mathbf{a}' = X_{\mathrm{R}}\,\mathbf{a}$ is the new basis and $D' = DX_{\mathrm{R}}^{\mathrm{T}}$ represents the transformed mode-port coupling matrix. In the new eigenmode basis, the evolution matrix Θ' is defined as:

$$\Theta' = X_{\mathrm{R}}\,\Theta X_{\mathrm{R}}^{\mathrm{T}}. \tag{40}$$

Given the fact that Θ is symmetric, Supplementary Equations 34 and 35 directly imply that Θ' is diagonal. Therefore, without loss of generality one can consider the optical evolution matrix as follows:

$$\Theta = \begin{pmatrix} \Delta_1 & 0 \\ 0 & \Delta_2 \end{pmatrix} + \mathrm{i}/2 \begin{pmatrix} \kappa_1 & 0 \\ 0 & \kappa_2 \end{pmatrix}. \tag{41}$$

Supplementary Note 5: Derivation of the determinant of the coupling matrix D

As shown in Supplementary Equation 26, the contrast between forward and backward transmission coefficients is directly proportional with the determinant of the mode-port coupling matrix D thus breaking the reciprocity demands $\det(D) \neq 0$. Here, we show that this determinant can in general be obtained in terms of the out-coupling losses of the optical modes. In fact, the energy conservation relation $D^\dagger D = K_\mathrm{e}$ implies that:

$$|\det(D)|^2 = \det(K_\mathrm{e}) = \eta_1 \kappa_1 \eta_2 \kappa_2, \tag{42}$$

where $\eta_1 = \frac{\kappa_{\mathrm{e}_1}}{\kappa_1}$ and $\eta_2 = \frac{\kappa_{\mathrm{e}_2}}{\kappa_2}$ represent the ratio of out-coupling to total losses for each mode. On the other hand, the time reversal symmetry requirement of the optical system, i.e., $CD^* = -D$ imposes a condition on the phase of this determinant according to $\det(C)\det(D)^* = \det(D)$. Multiplying left and right with $\det(D)$ yields $\det(C)|\det(D)|^2 = (\det(D))^2$. Supplementary Equation 42 now directly gives

$$\det(D) = \sqrt{\eta_1 \eta_2 \kappa_1 \kappa_2 \det C}. \tag{43}$$

Note that in general, C is a unitary matrix with $|\det(C)| = 1$. In addition, by adjusting the evaluation point at the two ports one can properly choose the phase of $\det(C)$. For the system we consider in Supplementary Note 6, we have chosen $\det(C) = -1$, which results in $\det(D) = \mathrm{i}\sqrt{\eta_1 \eta_2 \kappa_1 \kappa_2}$.

According to this relation a necessary condition for breaking the reciprocity is that $\eta_1 \neq 0$ and $\eta_2 \neq 0$. This latter means that both optical modes should be coupled to the ports, or equivalently, the number of independent decay ports (l=rank(D)) should be equal to the number of the actual ports. A possible scenario that violates this condition is the presence of a dark state which is decoupled from the two ports of the system [6]. This is in fact a scenario that would arise when diagonalizing a system that consists of two modes that have equal symmetry with respect to the output channels, through which they would couple at finite rate κ_r.

Supplementary Note 6: Microring resonator system

Here we explore the microring resonator as a specific case of the general formalism derived in previous sections. In such structure, the direct scattering matrix C can be written as:

$$C = \begin{pmatrix} 0 & 1 \\ 1 & 0 \end{pmatrix}, \tag{44}$$

Here, instead of using the clockwise a_cw and counterclockwise a_ccw traveling modes, we consider the even and odd standing modes, $a_1 = (a_\mathrm{cw} + a_\mathrm{ccw})/\sqrt{2}$ and $a_2 = (a_\mathrm{cw} - a_\mathrm{ccw})/\mathrm{i}\sqrt{2}$, as our base modes. We consider a potentially non-zero frequency splitting $\delta\omega_2 - \delta\omega_1$ between the two even and odd modes. Therefore, the Θ matrix involving resonance frequencies can be written as in Supplementary Equation 18. On the other hand, the symmetry/anti-symmetry of the even/odd mode directly implies [1] $d_{11} = d_{21}$ and $d_{12} = -d_{22}$. The time reversal symmetry requirement, $CD^* = -D$, therefore leads to $d_{11}^* = -d_{11}$ and $d_{22}^* = d_{22}$. While the power conservation relation, $D^\dagger D = K_\mathrm{e}$ leads to $\kappa_{\mathrm{e}_1} = 2|d_{11}|^2, \kappa_{\mathrm{e}_2} = 2|d_{22}|^2$, thus D is obtained as

$$D = \frac{1}{\sqrt{2}} \begin{pmatrix} \mathrm{i}\sqrt{\kappa_{\mathrm{e}_1}} & -\sqrt{\kappa_{\mathrm{e}_2}} \\ \mathrm{i}\sqrt{\kappa_{\mathrm{e}_1}} & \sqrt{\kappa_{\mathrm{e}_2}} \end{pmatrix}. \tag{45}$$

Based on Supplementary Equations 5 and 45, the scattering matrix is written as

$$S = \begin{pmatrix} 0 & 1 \\ 1 & 0 \end{pmatrix} + \frac{\mathrm{i}}{2} \begin{pmatrix} \mathrm{i}\sqrt{\kappa_{\mathrm{e}_1}} & -\sqrt{\kappa_{\mathrm{e}_2}} \\ \mathrm{i}\sqrt{\kappa_{\mathrm{e}_1}} & \sqrt{\kappa_{\mathrm{e}_2}} \end{pmatrix} \begin{pmatrix} \Sigma_{\mathrm{o}_1} \mp \frac{|g_1|^2}{\Sigma_{\mathrm{m}}^{\pm}} & \mp \frac{g_1 g_2^*}{\Sigma_{\mathrm{m}}^{\pm}} \\ \mp \frac{g_1^* g_2}{\Sigma_{\mathrm{m}}^{\pm}} & \Sigma_{\mathrm{o}_2} \mp \frac{|g_2|^2}{\Sigma_{\mathrm{m}}^{\pm}} \end{pmatrix}^{-1} \begin{pmatrix} \mathrm{i}\sqrt{\kappa_{\mathrm{e}_1}} & \mathrm{i}\sqrt{\kappa_{\mathrm{e}_1}} \\ -\sqrt{\kappa_{\mathrm{e}_2}} & \sqrt{\kappa_{\mathrm{e}_2}} \end{pmatrix}. \tag{46}$$

From here, the forward and backward transmission coefficients are obtained as follows:

$$S_{12} = 1 - \frac{\mathrm{i}}{2} \frac{\kappa_{\mathrm{e}_2}(\Sigma_{\mathrm{o}_1}\Sigma_{\mathrm{m}}^{\pm} \mp |g_1|^2) + \kappa_{\mathrm{e}_1}(\Sigma_{\mathrm{o}_2}\Sigma_{\mathrm{m}}^{\pm} \mp |g_2|^2) \mp \mathrm{i}\sqrt{\kappa_{\mathrm{e}_1}\kappa_{\mathrm{e}_2}}(g_1 g_2^* - g_1^* g_2)}{\Sigma_{\mathrm{m}}^{\pm}\Sigma_{\mathrm{o}_1}\Sigma_{\mathrm{o}_2} \mp \Sigma_{\mathrm{o}_2}|g_1|^2 \mp \Sigma_{\mathrm{o}_1}|g_2|^2}, \tag{47}$$

$$S_{21} = 1 - \frac{\mathrm{i}}{2} \frac{\kappa_{\mathrm{e}_2}(\Sigma_{\mathrm{o}_1}\Sigma_{\mathrm{m}}^{\pm} \mp |g_1|^2) + \kappa_{\mathrm{e}_1}(\Sigma_{\mathrm{o}_2}\Sigma_{\mathrm{m}}^{\pm} \mp |g_2|^2) \pm \mathrm{i}\sqrt{\kappa_{\mathrm{e}_1}\kappa_{\mathrm{e}_2}}(g_1 g_2^* - g_1^* g_2)}{\Sigma_{\mathrm{m}}^{\pm}\Sigma_{\mathrm{o}_1}\Sigma_{\mathrm{o}_2} \mp \Sigma_{\mathrm{o}_2}|g_1|^2 \mp \Sigma_{\mathrm{o}_1}|g_2|^2}. \tag{48}$$

These latter relations can be rewritten in terms of multi-photon cooperativities of both modes as follows:

$$S_{12} = 1 - \mathrm{i}\frac{\eta_2\left((\delta_1 + \mathrm{i})(\delta_{\pm} + \mathrm{i}) \mp \mathcal{C}_1\right) + \eta_1\left((\delta_2 + \mathrm{i})(\delta_{\pm} + \mathrm{i}) \mp \mathcal{C}_2\right) \mp 2\sqrt{\eta_1\eta_2}\sqrt{\mathcal{C}_1\mathcal{C}_2}\sin(\Delta\phi)}{(\delta_1 + \mathrm{i})(\delta_2 + \mathrm{i})(\delta_{\pm} + \mathrm{i}) \mp (\delta_2 + \mathrm{i})\mathcal{C}_1 \mp (\delta_1 + \mathrm{i})\mathcal{C}_2}, \tag{49}$$

$$S_{21} = 1 - \mathrm{i}\frac{\eta_2\left((\delta_1 + \mathrm{i})(\delta_{\pm} + \mathrm{i}) \mp \mathcal{C}_1\right) + \eta_1\left((\delta_2 + \mathrm{i})(\delta_{\pm} + \mathrm{i}) \mp \mathcal{C}_2\right) \pm 2\sqrt{\eta_1\eta_2}\sqrt{\mathcal{C}_1\mathcal{C}_2}\sin(\Delta\phi)}{(\delta_1 + \mathrm{i})(\delta_2 + \mathrm{i})(\delta_{\pm} + \mathrm{i}) \mp (\delta_2 + \mathrm{i})\mathcal{C}_1 \mp (\delta_1 + \mathrm{i})\mathcal{C}_2}. \tag{50}$$

Derivation of D matrix in a quantum optics approach

Supplementary Note 2 showed the transformation between the formalisms of coupled mode theory and a standard quantum optics input-output theory. Here we briefly demonstrate that the matrix D can alternatively be obtained from input-output theory. We will start by considering the mode basis of propagating (clockwise and counterclockwise) modes, captured in the vector $\mathbf{a}_{\mathrm{rot}} = (a_{\mathrm{cw}}, a_{\mathrm{ccw}})^{\mathrm{T}}$, coupled to input fields $\mathbf{s}^-$ and output fields $\tilde{\mathbf{s}}^+$. Note that in this case the jth component of $\tilde{\mathbf{s}}^+$ corresponds to a wave in a different physical waveguide than the jth component of $\mathbf{s}^-$, as $\tilde{\mathbf{s}}^+ = C\mathbf{s}^+$. In this basis, the equations of motion read

$$\dot{\mathbf{a}}_{\mathrm{rot}} = \mathrm{i}M_{\mathrm{rot}}\mathbf{a}_{\mathrm{rot}} - D_{\mathrm{rot}}^{\dagger}\tilde{\mathbf{s}}^+, \tag{51}$$

where $D_{\mathrm{rot}} = \sqrt{\kappa_{\mathrm{e}}}I$ and

$$M = \begin{pmatrix} -\omega_{\mathrm{c}} + \mathrm{i}\frac{\kappa}{2} & \gamma \\ \gamma & -\omega_{\mathrm{c}} + \mathrm{i}\frac{\kappa}{2} \end{pmatrix}, \tag{52}$$

where γ is a (potentially complex) coupling rate that accounts for possible mode splitting.

We can transform to a basis of even and odd modes, captured in the vector $\mathbf{a} = (a_1, a_2)^{\mathrm{T}}$ via the unitary transformation $\mathbf{a} = U\mathbf{a}_{\mathrm{rot}}$, where

$$U = \frac{1}{\sqrt{2}} \begin{pmatrix} 1 & 1 \\ -\mathrm{i} & \mathrm{i} \end{pmatrix}. \tag{53}$$

The equation of motion for $\mathbf{a}$ can then be written in the familiar form

$$\dot{\mathbf{a}} = \mathrm{i}M\mathbf{a} - D^{\dagger}\tilde{\mathbf{s}}^+ \tag{54}$$

by defining

$$M = UM_{\rm rot}U^{-1} = \begin{pmatrix} -\omega_{\rm c} + {\rm i}\frac{\kappa}{2} + \gamma & 0 \\ 0 & -\omega_{\rm c} + {\rm i}\frac{\kappa}{2} - \gamma \end{pmatrix} \tag{55}$$

and $D^\dagger = UD^\dagger_{\rm rot}$ such that

$$D = \frac{-{\rm i}}{\sqrt{2}} \begin{pmatrix} {\rm i}\sqrt{\kappa_{\rm e}} & -\sqrt{\kappa_{\rm e}} \\ {\rm i}\sqrt{\kappa_{\rm e}} & \sqrt{\kappa_{\rm e}} \end{pmatrix}. \tag{56}$$

As expected from the discussion in Supplementary Note 2, the derived matrix D is identical to that obtained above (Supplementary Equation 45) up to an overall phase factor $-{\rm i}$ that can be absorbed in the definition of the intracavity fields. Note that in Supplementary Equation 45 we have explicitly included the possibility of a difference in the coupling of the two orthogonal modes to the waveguide.

Propagation direction of the intracavity field

The D matrix derived in this section can also be used to determine the relative phase with which an incident probe beam drives the modes a_1 and a_2, which determines the handedness of the intracavity field. Consider the following expression that is used to determine the field in the modes a_1 and a_2

$$\begin{pmatrix} a_1 \\ a_2 \end{pmatrix} = \begin{pmatrix} \Sigma_{\rm o_1}^{-1}(\omega) & 0 \\ 0 & \Sigma_{\rm o_2}^{-1}(\omega) \end{pmatrix} D^{\rm T} \begin{pmatrix} \bar{s}_{\rm in} \\ 0 \end{pmatrix} = -\frac{\bar{s}_{\rm in}}{\sqrt{2}} \begin{pmatrix} \frac{2}{\kappa_1}\frac{\sqrt{\eta_1\kappa_1}}{\delta_1+{\rm i}} \\ {\rm i}\frac{2}{\kappa_2}\frac{\sqrt{\eta_2\kappa_2}}{\delta_2+{\rm i}} \end{pmatrix}, \tag{57}$$

where we used the D matrix given by Supplementary Equation 45. If the modes are degenerate (such that $\delta_1 = -\delta_2 = 0$) and have equal loss rates, it follows from Supplementary Equation 57 that a_2 is driven with an additional phase shift of $\pi/2$ with respect to a_1. In such a situation, the intracavity field will propagate in the same direction as the incident probe field. On the other hand, when the modes are split ($\delta_1 \gg 1$), the phase relation between a_1 and a_2 is reversed. Importantly, this means that the intracavity probe field is then no longer a wave propagating in the same direction as the incident probe field. In the presence of strong mode splitting, a probe beam that propagates opposite of the control beam in the waveguide thus induces an intracavity field that propagates along with the control beam. In regime of modest mode splitting, as experimentally shown in the manuscript, both probe beams couple to the mechanical mode. Crucially, the fact the control fields of even and odd modes are always $\sim \pi/2$ out of phase in this setup guarantees maximum isolation for any splitting, in line with Eq. (10) in the manuscript.

Optical modes with identical loss and coupling

In order to explore the maximum contrast between the forward and backward transmission coefficients, we consider a resonant probe excitation corresponding to $\omega = \pm\Omega_{\rm m}$. Furthermore, the two optical modes exhibit the same amount of losses ($\kappa_{{\rm e}_{1,2}} = \kappa_{\rm e}$, $\kappa_{0_{1,2}} = \kappa_0$), and for simplicity we also assume that both modes are pumped with the same internal cavity photon numbers ($|g_{1,2}| = |g|$) while they can in general exhibit different phases. In this case the scattering parameters can be greatly simplified to:

$$S_{12} = 1 - \eta\frac{2 \pm \mathcal{C}(1 + \sin(\Delta\phi))}{1 \pm \mathcal{C} + \beta^2}, \tag{58}$$

$$S_{21} = 1 - \eta\frac{2 \pm \mathcal{C}(1 - \sin(\Delta\phi))}{1 \pm \mathcal{C} + \beta^2}, \tag{59}$$

where $\mathcal{C} = 2\mathcal{C}_1 = 2\mathcal{C}_2$ and $\beta = (\omega + \bar{\Delta}_1)/(\kappa/2) = -(\omega + \bar{\Delta}_2)/(\kappa/2)$. Note that both transmissions are real quantities and their contrast is obtained as

$$S_{21} - S_{12} = 2\eta \frac{\pm\mathcal{C}\sin(\Delta\phi)}{1 \pm \mathcal{C} + \beta^2}, \tag{60}$$

This relation clearly shows that maximum contrast can be obtained for $\Delta\phi = \pi/2$, i.e., $g_2 = \mathrm{i}g_1$. Under the condition of critical coupling ($\eta = \kappa_\mathrm{e}/\kappa = 1/2$) the difference between the transmittivities of the forward and backward probes is obtained from

$$\Delta T = |S_{21}|^2 - |S_{12}|^2 = \frac{\mathcal{C}(\mathcal{C} \pm 2\beta^2)}{(1 \pm \mathcal{C} + \beta^2)^2}, \tag{61}$$

which is similar to equation (11) in the main text and can be further simplified to $\Delta T = \mathcal{C}^2/(\mathcal{C} \pm 1)^2$ for the case of degenerate modes. This latter relation shows that the contrast between forward and backward transmission coefficients is monotonically increasing with cooperativity and asymptotically approaches unity for $\mathcal{C} \to \infty$. In addition, it is possible to properly adjust a finite $\mathcal{C}$ so that the backward probe can be completely blocked, i.e., $S_{12} = 0$. From Supplementary Equation 59, for the red detuned regime, such relation is obtained to be:

$$(2\eta - 1)(1 + \mathcal{C}) = \beta^2 \tag{62}$$

Note that this relation can only be satisfied for a strongly coupled system, i.e., when $\eta > 1/2$. For zero mode splitting ($\beta = 0$) Supplementary Equation 62 reduces to the condition of critical coupling $\eta = 1/2$. In such a scenario, independent from the cooperativity, the backward probe can always be fully blocked, while larger cooperativities can increase the forward transmission.

Supplementary References

[1] Suh, W., Wang, Z. & Fan, S. Temporal coupled-mode theory and the presence of non-orthogonal modes in lossless multimode cavities. *IEEE J. Quant. Electron.* **40**, 1511–1518 (2004).

[2] Collett, M. J. & Gardiner, C. W. Squeezing of intracavity and traveling-wave light fields produced in parametric amplification. *Phys. Rev. A* **30**, 1386–1391 (1984).

[3] Gardiner, C. & Zoller, P. *Quantum noise: a handbook of Markovian and non-Markovian quantum stochastic methods with applications to quantum optics* (Springer, 2004), 3rd edn.

[4] Walls, D. F. & Milburn, G. J. *Quantum Optics* (Springer, 2008), 2nd edn.

[5] Arfken, G. *Mathematical Methods for Physicists* (Academic Press, 1985), 3rd edn.

[6] Gentry, C. M. & Popović, M. A. Dark state lasers. *Opt. Lett.* **39**, 4136–4139 (2014).
